# Epidemiological trends and age-period-cohort effects on intracerebral hemorrhage burden across the BRICS-plus from 1992 to 2021

**DOI:** 10.3389/fneur.2025.1575324

**Published:** 2026-01-12

**Authors:** Fangqun Cheng, Yuhang Wu, Shudong Xie, Yan Wang, Caihong Chen, Limin Song, Ling Zhu, Miaozhen Wang, Wei Mo, Xiaochan Wang

**Affiliations:** 1 Xiangtan Central Hospital, Xiangtan, China; 2 Department of Epidemiology and Health Statistics, Xiangya School of Public Health, Central South University, Changsha, China; 3 Transplantation Center, The Third Xiangya Hospital, Central South University, Changsha, Hunan, China; 4 Hunan Provincial People's Hospital, The First Affiliated Hospital of Hunan Normal University, Changsha, Hunan, China

**Keywords:** age-period-cohort model, BRICS, disability-adjusted life years, intracerebral hemorrhage, public health

## Abstract

**Background:**

Intracerebral hemorrhage (ICH) is a major global health concern associated with significant morbidity and mortality. The BRICS nations—comprising Brazil, Russian Federation, India, China, and South Africa—along with an expansion to include Saudi Arabia, Egypt, United Arab Emirates, Iran, and Ethiopia (BRICS-plus), represent a significant proportion of the global population and present unique health challenges. This study aims to evaluate the epidemiological trends and variations in the burden of ICH across BRICS-plus nations in a timely manner.

**Methods:**

Data on the number, all-age rate, age-standardized rate, and relative change in ICH disability-adjusted life years (DALYs) from 1992 to 2021 within BRICS-plus were obtained from the Global Burden of Disease Study (GBD) 2021. Relationships between the DALYs rate and the Socio-demographic Index (SDI) were evaluated using Pearson correlation analyses. Additionally, age-period-cohort modeling was employed to estimate net drift, local drift, age, period, and cohort effects over the past three decades.

**Results:**

All BRICS-plus countries exhibited a declining trend in the age-standardized DALYs rate from 1992 to 2021. China reported the highest DALYs rate (1351.55 per 100,000 population) in 2021, while Brazil displayed the most significant decrease, at 64.53%. The annual net drift in the ICH DALYs rate ranged from −3.99% for Ethiopia to −1.61% for India among the ten countries. A significant negative correlation was observed between the DALYs rate of ICH and SDI values. Countries exhibited similar age effect patterns, with an increasing risk of DALYs rate with advancing age, as well as varying period and cohort effects, indicative of differential control measures and temporal burden trends.

**Conclusion:**

The burden of ICH showed an overall declining trend across the BRICS-plus from 1992 to 2021, but persistent health inequalities between these countries were driven by socioeconomic disparities. Furthermore, it emphasizes the necessity for targeted interventions across age, period, and cohort dimensions to address the distinct challenges posed by ICH in these rapidly developing countries.

## 1 Introduction

Intracerebral hemorrhage (ICH), a subtype of stroke, poses a significant global health challenge due to its high morbidity and mortality rates (1, 2). This condition arises when a cerebral blood vessel ruptures, leading to localized hemorrhage and neuronal damage. Given the limited efficacy of current therapeutic interventions, the prevention and management of ICH remain critical priorities in public health. Epidemiologically, ICH constitutes approximately 28% of all strokes but disproportionately contributes to stroke-related disabilities and mortality (3). Global estimates suggest that ICH imposes substantial social and economic burdens, both by causing immediate life-threatening conditions and long-term disabilities requiring extensive rehabilitation and support (4–6). A timely and comprehensive assessment of the growing ICH burden is essential for developing effective, locally tailored healthcare policies and preventive strategies to mitigate its severe public health impacts.

According to the World Health Organization (WHO) Director General Margaret Chan, Brazil, Russian Federation, India, China, and South Africa (BRICS) not only account for 43% of the world’s population, but also, “represent a block of countries with a fresh and invigorating approach to global health (7).” Effective January 1, 2024, BRICS will expand to include Saudi Arabia, Egypt, the United Arab Emirates, Iran, and Ethiopia, thereby broadening its scope to 10 nations (BRICS-plus). This enlargement underscores the collective geopolitical and socio-economic importance of these nations. Given their significant and growing contribution to the global disease burden, an extensive investigation of the epidemiological characteristics of neurological disorders, particularly ICH, within the evolving dynamics of the BRICS-plus nations, is imperative. Previous research using data from the Global Burden of Disease (GBD) 2019 provided initial insights into the geographic heterogeneity in the burden of ICH (3, 8). These studies have revealed significant disparities in health metrics between and within nations, particularly within developing regions. Evidence-based healthcare decision-making at both regional and national levels remains essential. However, these traditional descriptive approaches are often limited in their comprehensive understanding of underlying temporal trends and demographic influences. A compelling analytical framework is provided by the Age-Period-Cohort (APC) model (9), which offers enhanced precision for disentangling the intricate relationships between age, period, and cohort effects on ICH epidemiology. Moreover, the recently updated GBD 2021 dataset offers an unparalleled chance to explore the trending burden and spatiotemporal trajectories of ICH within the expanded BRICS sphere (10–12). The utilization of data from GBD 2021 enables critical surveillance, necessary for health policy formulation and implementation targeted at mitigating stroke-related health adversities.

To address these gaps, we conducted a comprehensive analysis of the ICH burden in BRICS-plus countries using data from the GBD 2021 study. Specifically, this study aimed to (i) characterize long-term temporal trends and between-country differences in disability-adjusted life years (DALYs) due to ICH from 1992 to 2021; (ii) examine the association between age-standardized ICH DALY rates and the Socio-demographic Index (SDI) across BRICS-plus nations; and (iii) disentangle age, period, and cohort effects on ICH DALYs in each country in order to inform more targeted prevention and control strategies.

## 2 Methods

### 2.1 Data sources

This study used data from the GBD 2021 public dataset, available through the Global Health Data Exchange GBD Results Tool.^1^ The GBD 2021 dataset offers comprehensive insights into the disease burden of 371 conditions across 204 countries and territories worldwide (10–13). It is an extensive resource that includes a wide range of health-related data on disease burden, risks, mortality, and disability, providing a vital repository for understanding global health issues. In particular, GBD 2021 featured several new enhancements, such as the addition of 19,189 DALYs data sources, the inclusion of data for 12 new health conditions, and various methodological advancements. Moreover, it considered the impact of the COVID-19 pandemic on the global burden of disease.

For ICH, the GBD 2021 follows the definitions set out by the International Classification of Diseases, 9th edition (ICD-9 codes: 431, 431.1–432.9), and 10th edition (ICD-10 codes: I61-I62, I62.9, I69.0-I69.298) (13). The study obtained the number of DALYs, all-age rates, and age-standardized rates of DALYs due to ICH globally and across 10 BRICS-plus countries. DALYs are calculated to assess the overall burden of disease by combining the years of life lost due to premature mortality with the years lived with disability, providing a comprehensive measure of the impact on public health. Data was collected across age groups 0 to 94 years, spanning the period from 1992 to 2021. Reflecting the GBD database’s characteristics in model selection, parameter estimation, and data quality and availability, the 95% uncertainty intervals (UIs) were obtained by replicating the sample 1,000 times, with upper and lower bounds identified through the 2.5th and 97.5th percentiles of the uncertainty distribution (10). Additionally, the GBD 2021 updated the SDI for BRICS-plus countries. The methodologies and modeling approach for GBD 2021 has been documented in other publications (10–13). This data was anonymized and made publicly available, and the study protocol, which has a waiver for informed consent, was approved by the University of Washington Institutional Review Board.

### 2.2 Data analysis

#### 2.2.1 Analysis of overall temporal trends in ICH DALYs

Temporal trends in DALYs over the study period were assessed by the number, all-age DALY rate, age-standardized DALY rate, and the relative percentage change from 1992 to 2021. We then assessed the relationship between SDI values of 10 countries and age-standardized rates of DALYs by using Pearson correlation coefficient. Furthermore, the age distribution of the ICH burden was examined by categorizing the number of DALYs into five age strata (0–4, 5–19, 20–39, 40–64, and 65–94 years) and calculating the proportion of DALYs in each age stratum.

#### 2.2.2 Age period cohort modeling analysis

The APC model is a statistical tool based on the Poisson distribution, which is used to extract and reveal potential insights about disease trends (14, 15). In this study, we applied the APC model to decompose the DALYs into three key dimensions: age, period, and birth cohort, subsequently analyzing their respective impacts on ICH DALYs (14, 15). Within this model, age effects indicate the varying risks of the outcome for different age groups. Period effects capture temporal changes that impact all age groups simultaneously, whereas cohort effects highlight outcome variations for individuals born in the same year. The formulation of the APC model can be expressed as follows:


$$g(Y_{j}/\mu )=\log(\lambda_{j})=u+\alpha \;\mathrm{ag}e_{j}+\beta \;\text{perio}d_{j}+\gamma \;\text{cohor}t_{j}$$

Where $\lambda_{j}$ represents the response variable of the net effect on ICH DALYs rate for group j ; $Y_{j}$ and μ represent the number of DALYs and the population at risk, respectively. α , β , and γ represent the coefficients of age, period, and birth cohort of the APC model, respectively. u represents the intercept of the model.

The 2021 GBD DALYs estimates for ICH, alongside the population data from each country, were used as inputs for the APC model that implemented the intrinsic estimator (IE) method. In this study, the IE method was applied to resolve the problem of parameter indeterminacy generated by the APC model’s age, period, and cohort effects (16). Further methodological details are available in prior literature (16, 17). For this model to perform accurately, equal intervals for both age and period are required. Consequently, the population aged 0–94 years was divided into 19 age groups (0–4, 5–9, 90–94), each spanning 5-year intervals. To enhance temporal specificity and to avoid smoothing away short-term fluctuations (for example, potential perturbations around the COVID-19 pandemic and abrupt changes in data collection), DALYs and population counts were centered on the mid-year point of six discrete periods (1994, 1999, 2004, 2009, 2014, and 2019) rather than five-year averages. This input data included 19 age groups and 21 continuous cohorts defined by birth intervals 1900 to 1904 (median 1902) to 2015–2019 (median 2017). As a sensitivity analysis, we repeated all APC models using conventional 5-year averaged DALYs and population data for each period.

In this study, we primarily focus on the following estimable functions (15). The net drift represents the overall annual percentage change for DALY rates over time. Local drifts reflect annual percentage changes by period and cohort for each age group, while the longitudinal age curve indicates the fitted longitudinal age-specific rates adjusted for period deviations within the reference cohort. The period (or cohort) rate ratio (RR) refers to the ratio of age-specific rates in each period (or cohort) relative to the reference one. For parameters estimated from our own models (e.g., net drift, local drifts, and relative risks of age, period and cohort effects), we present 95% confidence intervals (CI) based on standard normal approximations to the sampling distribution of the regression coefficients. APC analyses were conducted using the National Cancer Institute’s age-period-cohort web-based tool,^2^ with subsequent data visualization and statistical analysis performed in R (version 4.2.3). Statistical significance for parameters was assessed using the Wald χ^2^ test, with all tests being two-tailed.

## 3 Results

### 3.1 DALYs of ICH trends from 1992 to 2021

**Figure 1:**
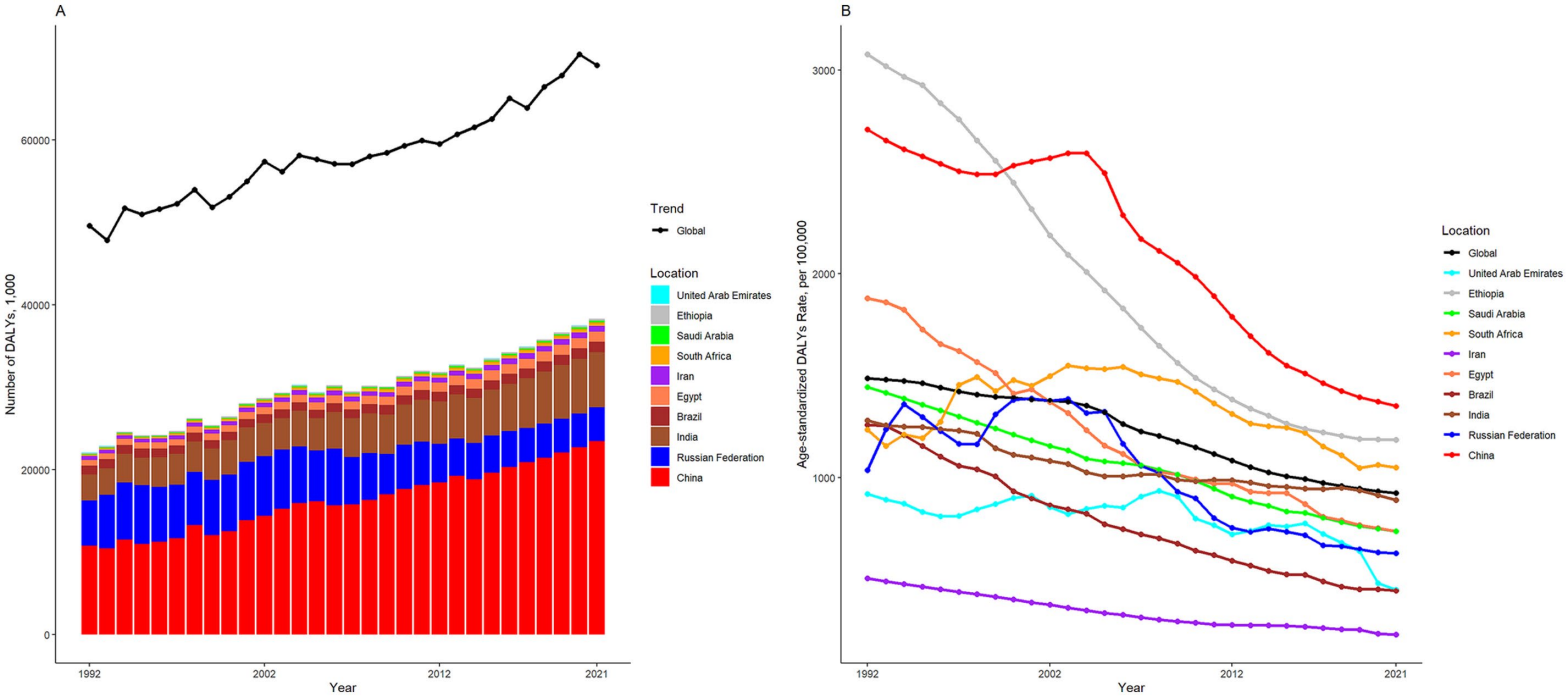
The numbers **(A)** and age-standardized rate **(B)** of DALYs of intracerebral hemorrhage in global and BRICS plus from 1992 to 2021. DALYs, disability-adjusted life years.

Table 1 presents the population, total DALYs, all-age DALYs rate, age-standardized DALYs rate, and net drift of DALYs for the world and ten BRICS-plus countries. Over the last thirty years, the number of DALYs attributable to ICH has increased from 64552.59 thousand (95% UI 60600.74 to 68787.34) in 1992 to 79457.43 thousand (95% UI 72748.91 to 85480.16) in 2021, marking a 23.09% rise (Table 1; Figure 1). Simultaneously, the age-standardized DALYs rate declined from 1486.32 (95% UI 1395.42 to 1587.72) per 100,000 population in 1992 to 923.64 (95% UI 844.83 to 993.18) per 100,000 population in 2021, indicating a 37.86% reduction (Table 1; Figure 1). According to the APC model, the net drift in the global ICH DALYs rate was −1.87% (95% CI −1.93 to −1.80) from 1992 to 2021 (Table 1).

**Table 1:** Trends in intracerebral hemorrhage disability-adjusted life years across the 10 BRICS countries, 1992–2021.

Characteristic	Global		Brazil		Russian federation		India		China		South Africa		Saudi Arabia		Egypt		United Arab Emirates		Iran		Ethiopia	
	1992	2021	1992	2021	1992	2021	1992	2021	1992	2021	1992	2021	1992	2021	1992	2021	1992	2021	1992	2021	1992	2021
Population																						
Number, *n* × 1,000,000^*^	5,497 (5,379, 5,624)	7,891 (7,668, 8,131)	154 (143, 166)	220 (188, 251)	152 (138, 166)	145 (125, 164)	888 (823, 960)	1,415 (1,240, 1,602)	1,213 (1,117, 1,309)	1,423 (1,319, 1,530)	39 (35, 42)	57 (50, 64)	17 (16, 18)	38 (33, 43)	58 (52, 63)	106 (96, 116)	2 (2, 2)	10 (8, 11)	60 (55, 66)	85 (77, 94)	55 (50, 59)	109 (92, 125)
Percentage of global, %	100.00	100.00	2.80	2.79	2.77	1.83	16.20	17.93	22.07	18.03	0.71	0.72	0.31	0.48	1.06	1.34	0.04	0.13	1.09	1.08	1.00	1.38
DALYs																						
Number, *n* × 1,000^*^	64552.59 (60600.74, 68787.34)	79457.43 (72748.91, 85480.16)	1352.20 (1319.41, 1385.96)	1135.06 (1081.88, 1181.02)	1905.20 (1854.79, 1942.59)	1393.90 (1291.39, 1501.59)	7017.00 (5883.20, 8111.69)	11098.17 (9239.87, 12841.99)	23002.59 (20122.67, 27166.18)	27463.75 (22839.24, 32676.71)	318.22 (284.55, 347.17)	513.34 (460.16, 559.19)	126.19 (100.86, 158.31)	206.49 (160.59, 263.01)	740.34 (493.74, 975.35)	516.11 (339.53, 692.52)	7.65 (6.02, 9.66)	18.96 (14.93, 23.11)	176.13 (158.17, 198.01)	186.75 (171.07, 203.51)	743.28 (583.36, 979.61)	568.47 (458.48, 688.99)
Percentage of global, %	100.00	100.00	2.23	1.79	11.53	5.87	6.56	9.50	21.84	33.30	0.39	0.58	0.22	0.31	1.45	1.80	0.02	0.04	0.91	0.97	0.25	0.28
Percent change of incidence 1992–2021, %	23.09		−16.06		−26.84		58.16		19.39		61.32		63.63		−30.24		147.84		6.03		−23.52	
All-age DALYs rate																						
Rate per 100,000^*^	1174.28 (1102.39, 1251.31)	1006.89 (921.88, 1083.21)	882.04 (860.66, 904.07)	515.10 (490.97, 535.96)	1255.51 (1222.29, 1280.15)	962.28 (891.51, 1036.62)	792.62 (664.55, 916.27)	784.60 (653.23, 907.89)	1906.97 (1668.21, 2252.14)	1930.33 (1605.29, 2296.74)	822.94 (735.87, 897.82)	902.91 (809.36, 983.55)	739.55 (591.10, 927.80)	547.66 (425.92, 697.55)	1287.35 (858.54, 1696.00)	488.61 (321.44, 655.62)	364.58 (287.07, 460.44)	196.87 (155.03, 239.95)	292.85 (262.99, 329.23)	218.79 (200.43, 238.43)	1354.36 (1062.97, 1784.99)	521.83 (420.86, 632.46)
Percent change of rate 1992–2021, %	−14.25		−41.60		−23.36		−1.01		1.22		9.06		−25.95		−62.05		−46.00		−25.29		−61.47	
Age-standardized DALYs rate																						
Rate per 100,000^*^	1486.32 (1395.42, 1587.72)	923.64 (844.83, 993.18)	1257.59 (1219.80, 1288.98)	446.04 (424.98, 464.27)	1036.09 (1006.68, 1057.48)	628.62 (582.10, 677.06)	1279.86 (1067.28, 1479.99)	888.62 (733.24, 1030.81)	2708.04 (2369.33, 3177.79)	1351.55 (1129.11, 1600.86)	1234.18 (1071.41, 1361.74)	1050.16 (942.10, 1143.69)	1443.33 (1138.63, 1790.56)	736.06 (597.14, 890.62)	1879.32 (1218.48, 2435.19)	735.75 (477.34, 992.07)	919.94 (722.75, 1165.94)	448.56 (370.04, 536.14)	506.55 (454.59, 554.04)	230.56 (210.89, 251.65)	3076.37 (2445.57, 3923.30)	1186.10 (956.81, 1451.49)
Percent change of rate 1992–2021, %	−37.86		−64.53		−39.33		−30.57		−50.09		−14.91		−49.00		−60.85		−51.24		−54.48		−61.44	
APC model estimates																						
Net drift of incidence rate, % per year^#^	−1.87 (−1.93, −1.80)		−3.73 (−3.83, −3.63)		−2.80 (−3.19, −2.42)		−1.61 (−1.81, −1.40)		−2.57 (−2.74, −2.41)		−1.76 (−2.20, −1.32)		−2.37 (−2.54, −2.21)		−3.35 (−3.58, −3.11)		−2.43 (−3.09, −1.77)		−2.38 (−2.52, −2.23)		−3.99 (−4.07, −3.91)	

All-age DALYs rate: crude DALYs rate.

Age-standardized DALYs rate is computed by direct standardization with global standard population in the GBD 2021.

Net drifts are estimates derived from the age-period-cohort model and denotes overall annual percentage change in DALYs rate, which captures the contribution of the effects from calendar time and successive birth cohorts.

* Parentheses for all GBD health estimate indicate 95% uncertainty intervals due to the inherent characteristics of model selection, parameter estimation, and the quality and availability of data inputs for GBD 2021.

# Parentheses for net drift indicate 95% confidence intervals.

APC, age-period-cohort; UI, uncertainty interval; CI, confidential interval; DALYs, disability-adjusted life years.

**Figure 2:**
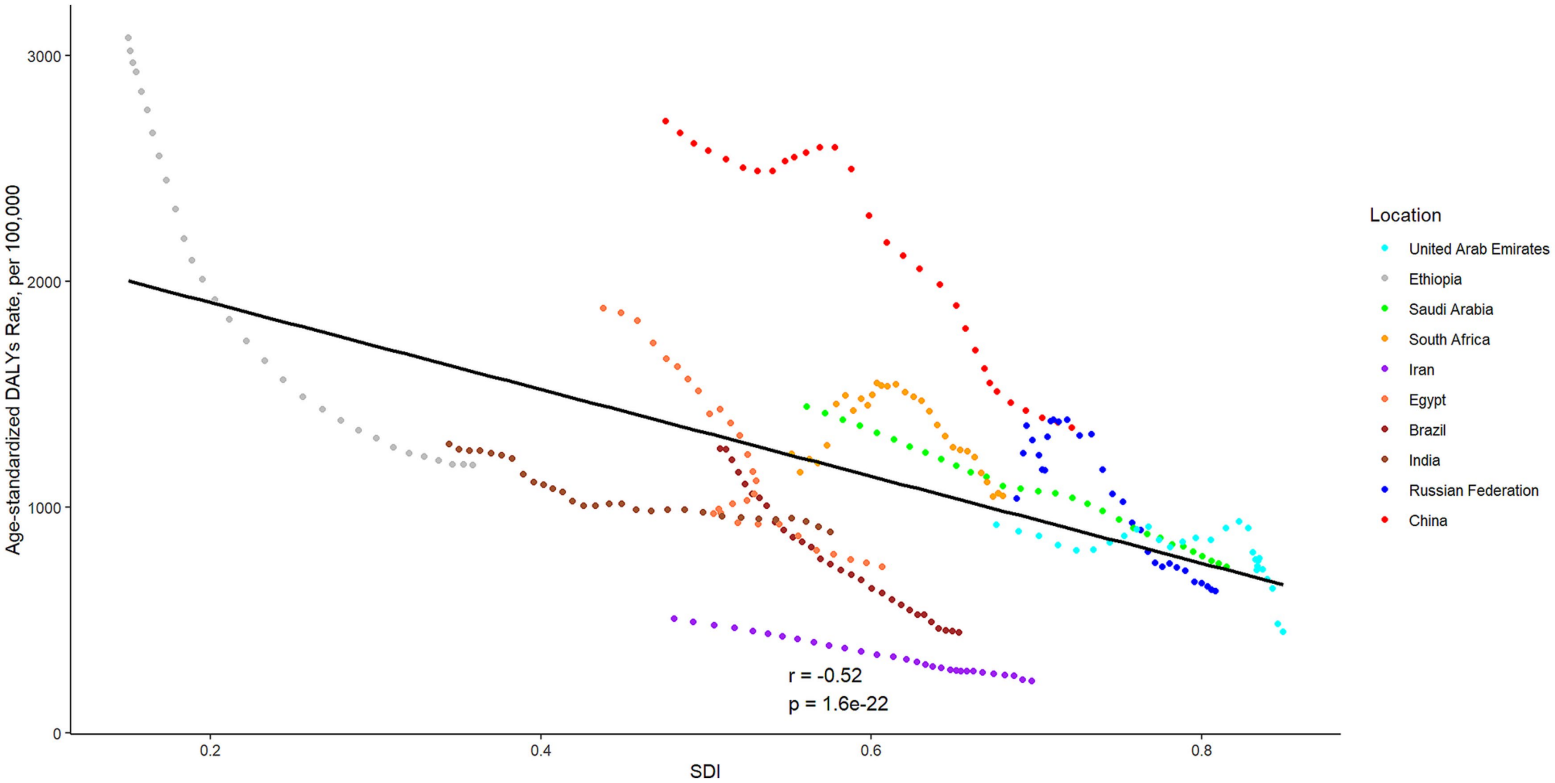
The associations between the SDI and age-standardized DALYs rates per 100,000 population of intracerebral hemorrhage across ten BRICS countries. SDI, Sociodemographic Index; DALYs, disability-adjusted life years.

The DALYs attributed to ICH were found to have significantly increased in six BRICS countries, specifically India, China, South Africa, Saudi Arabia, the United Arab Emirates, and Iran, with the United Arab Emirates demonstrating the most substantial increase at 147.84%. In 2021, the all-age DALYs rate and age-standardized DALYs rate for ICH varied considerably, from 218.79 (95% UI 200.43 to 238.43) and 230.56 (95% UI 210.89 to 251.65) per 100,000 population for Iran to 1930.33 (95% UI 1605.29 to 2296.74) and 1351.55 (95% UI 1129.11 to 1600.86) per 100,000 population in China, respectively. All BRICS-plus countries have exhibited a declining trend from 1992 to 2021. Among them, the most significantly observed rates were for Brazil, which displayed a decrease of 64.53%, while South Africa has shown the least significant decrease, at 14.91%. According to the APC model estimates, the annual net drift in the ICH DALYs rate ranged from −3.99% (95% CI −4.07 to −3.91) for Ethiopia to −1.61% (95% CI −1.81 to −1.40) for India within ten countries (Table 1). Furthermore, a significant negative correlation was observed between DALYs rate of ICH and SDI values (*r* = −0.52, *p* < 0.001) (Figure 2).

### 3.2 Time trends in ICH DALYs across different age groups

Supplementary Figure S1 depicts the annual percentage change in the DALYs rate of ICH across 5-year age groups, from 0 to 94 years. General speaking, all age groups exhibited negative local drift values, indicating a global decline in ICH DALYs rate. A similar pattern was observed in all BRICS-plus, with the exception of the United Arab Emirates. It should also be noted that females have more age groups (especially the older people) associated with positive net drift values than males in the United Arab Emirates. Supplementary Figure S2 illustrates the temporal trends in the number of DALYs for ICH by age group. Overall, the majority of global ICH DALYs were concentrated among the middle and older age groups (40 years and over), and comparable distributions were observed across original five members. In contrast, DALYs of ICH were distributed across all age groups in new BRICS countries. In Saudi Arabia, there was a notable shift, with the burden of DALYs moving from the older population (65–94 years) to younger and middle-aged groups (20–64 years).

### 3.3 Age, period, and cohort effects on ICH DALYs

**Figure 3:**
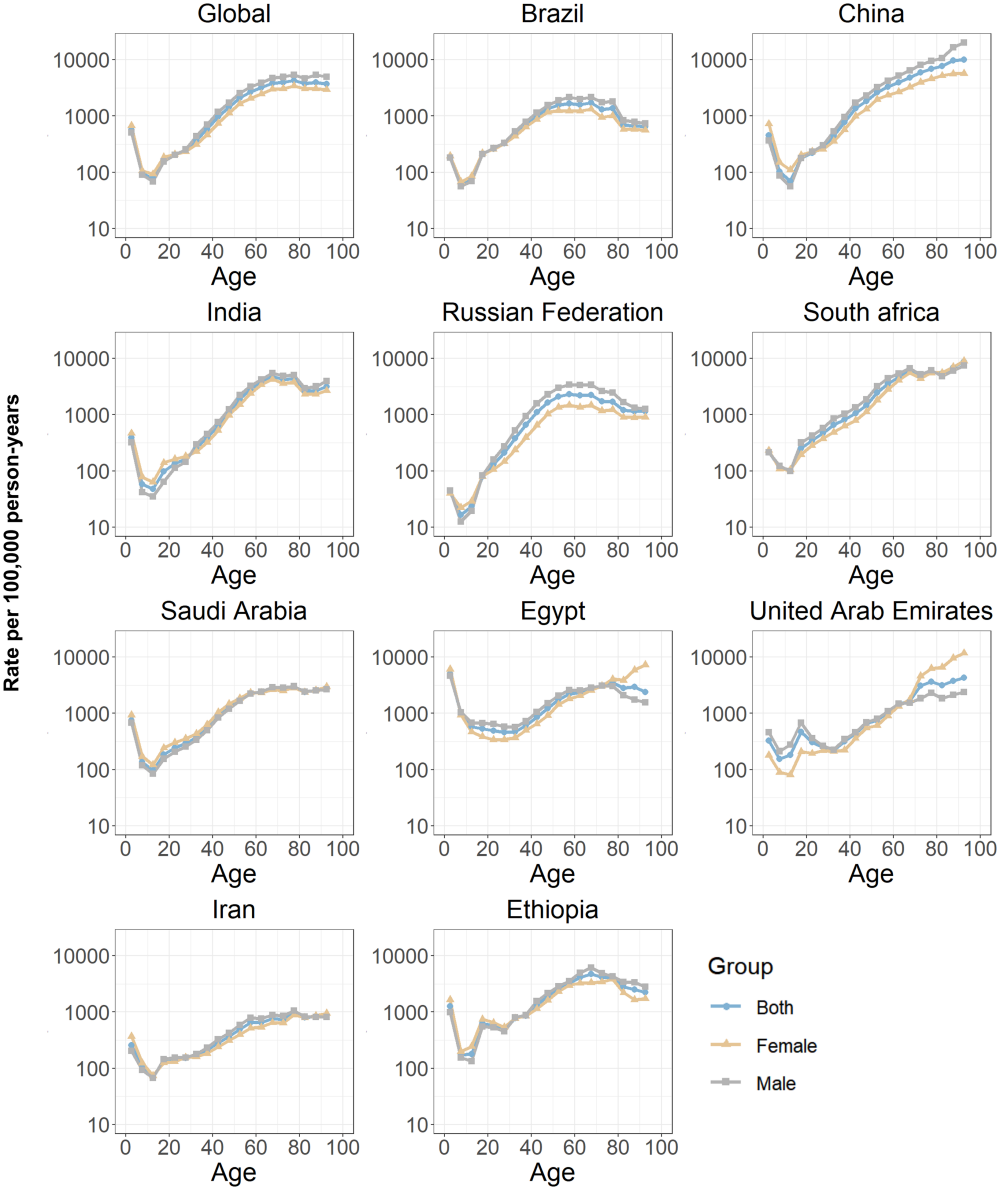
Age effects on intracerebral hemorrhage DALYs in global and BRICS plus. Age effects are shown by the fitted longitudinal age curves of DALYs rate (per 100,000 person-years) adjusted for period deviations. The dots denote DALYs rate. DALYs, disability-adjusted life years.

**Figure 4:**
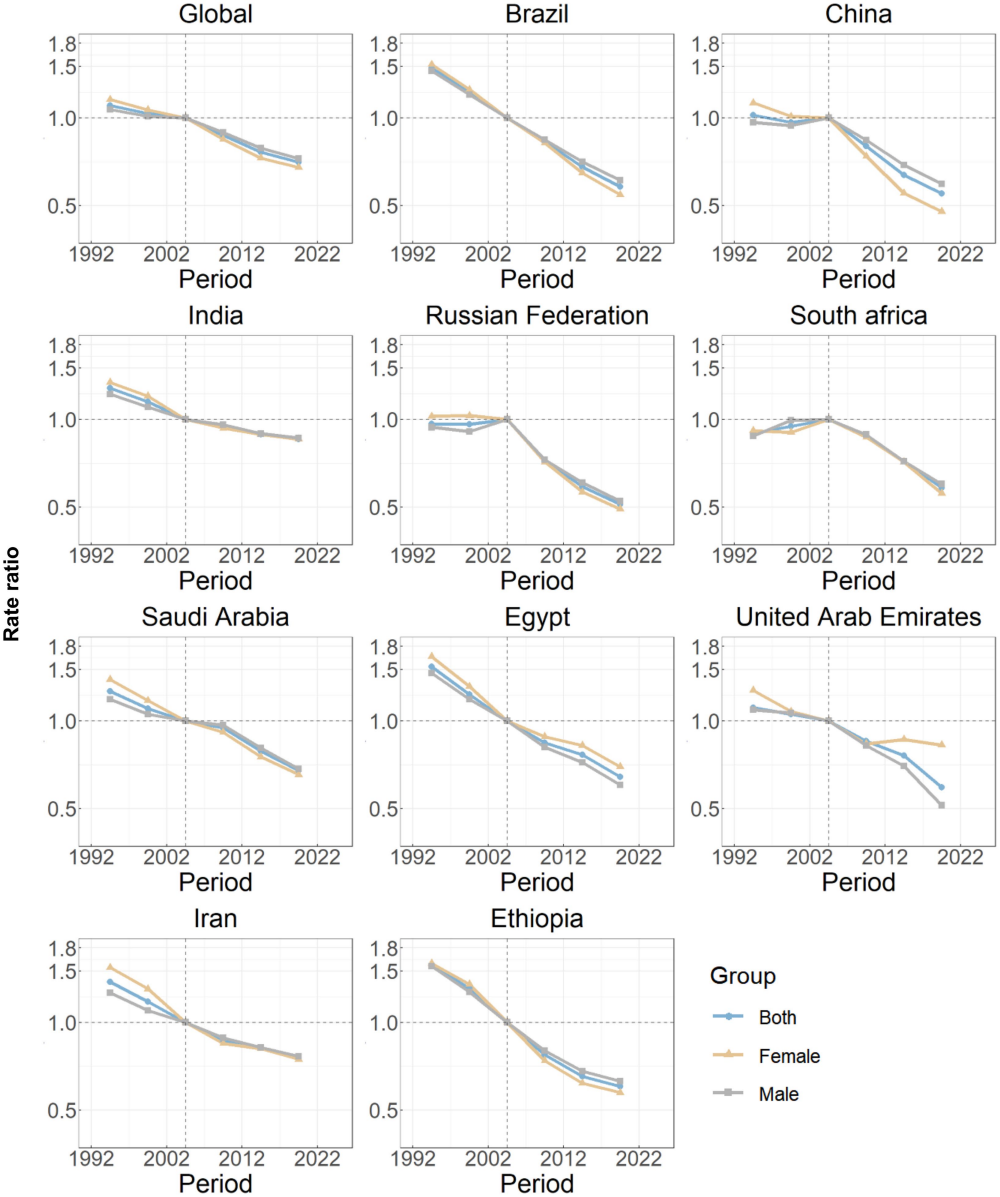
Period effects on intracerebral hemorrhage DALYs in global and BRICS plus. Period effects are shown by the relative risk of DALYs rate (DALYs rate ratio) and computed as the ratio of age-specific rates from 1992–1996 to 2017–2021, with the referent cohort set at 2002–2006. The dots denote DALYs rate ratios. DALYs, disability-adjusted life years.

**Figure 5:**
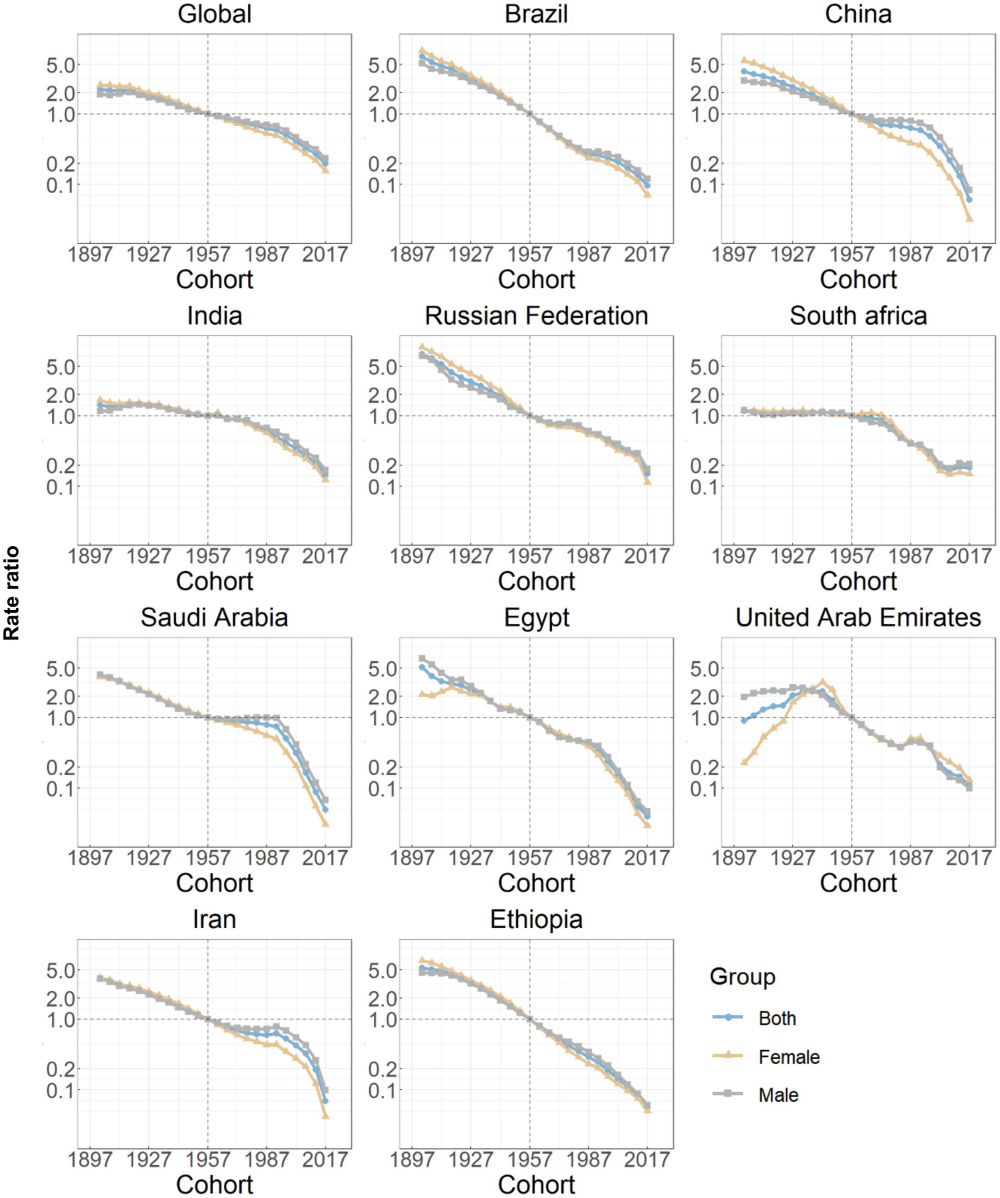
Cohort effects on intracerebral hemorrhage DALYs in global and BRICS plus. Cohort effects are shown by the relative risk of DALYs rate and computed as the ratio of age-specific rates from the 1902 cohort to the 2017 cohort, with the referent cohort set at 1957. The dots denote DALYs rate ratios. DALYs, disability-adjusted life years.

The estimates of the age effects derived from the APC model for global and BRICS-plus countries are illustrated by Figure 3. Overall, a consistent pattern of age effects was observed among all nations, in which the rate of DALYs attributed to ICH showed an upward trend with increasing age (10 years and over) in the reference cohort, adjusting for period effects. The estimated period effects by sex during the entire study period are shown in Figure 4. Period effects globally exhibited a continuous decline, which suggests effective control of ICH DALYs rates over time. Seven member countries (Brazil, India, Saudi Arabia, Egypt, the United Arab Emirates, Iran, and Ethiopia) demonstrate similar frameworks, with Brazil experiencing the most significant decline. China, the Russian Federation, and South Africa displayed relatively consistent period risks prior to the reference period (2002–2006), followed by a decrease. Cohort effects globally exhibited a continuous decline across successive birth cohorts over the past 50 years, as shown in Figure 5. This pattern was particularly evident in the majority of member countries (Brazil, China, the Russian Federation, Saudi Arabia, Egypt, the United Arab Emirates, Iran, and Ethiopia). In India and South Africa, the birth cohort RR initially remained relatively stable and subsequently began a decline. Additionally, in sensitivity analyses using five-year averaged DALY and population data for each period, the direction and magnitude of net drift, age effects, and the overall declining patterns of period and cohort rate ratios remained largely unchanged across countries (Supplementary Figures S3–S6), indicating that our findings are not materially driven by the chosen time-smoothing strategy.

## 4 Discussion

The study identifies marked fluctuations in the burden of ICH globally and within the BRICS-plus countries from 1992 to 2021. The analysis reveals a substantial 23.09% increase in the number of DALYs, while the age-standardized DALYs rate decreased by 37.86%, with notable variations among 10 BRICS members. Our findings further illuminate the multifaceted epidemiology of ICH and reflect striking health disparities, along with potential strategies for prioritization to alleviate its burden across age, period, and birth cohort dimensions within BRICS-plus.

The observed increase in the number of DALYs of ICH over the past three decades is likely driven by population growth. A marked heterogeneity in the DALYs rate and long-term trends of ICH exists across the BRICS-plus countries. Eight of 10 members show varying degrees of decrease in all-age DALYs rate, while China and South Africa show an increase. However, after adjusting for age distribution variations, changes in the pattern of age-standardized DALYs of ICH among the BRICS countries from 1992 to 2021 indicate that, beyond demographic shifts, other fundamental factors are influencing the observed changes in ICH burden. The vigorous development of neurological health programs and encouraging progress in medical and public health fields have been witnessed over the past three decades (18–20). Another aspect is that economic support is often considered essential for healthcare in cerebrovascular disease (3, 21), and our results may substantiate the validity of differentiating priority areas based on socioeconomic development. The significant negative correlation between DALYs rate of ICH and SDI underscores a multifaceted interplay involving early detection, preventative health measures, lifestyle improvements, and robust health frameworks facilitated by socio-demographic advancements (22, 23). However, the expression of this relationship is heterogeneous across BRICS-plus members. In upper-middle SDI countries such as Brazil, China and the Russian Federation, incremental gains in SDI have coincided with substantial reductions in age-standardized ICH DALY rates, likely mediated through expanded health-insurance coverage, strengthened primary care, higher educational attainment and declines in smoking prevalence. By contrast, in lower-SDI settings such as Ethiopia and, to a lesser extent, India and South Africa, improvements in SDI have been accompanied by more modest declines in ICH DALYs, reflecting persistent gaps in hypertension detection and treatment, uneven distribution of health workers and limited financial protection. These observations suggest that health-system capacity, population education and income security act as key mediating factors that determine how effectively socio-economic development can be translated into reductions in ICH burden.

Negative net drifts shown in almost observed countries and regions suggest overall improvements for medical technology, healthcare infrastructure, and increased public health initiatives targeting risk factors for cerebrovascular disease (24–26). In the United Arab Emirates, we observed positive local drift in several older female age groups, indicating a relative increase in ICH DALY rates over time compared with males. Although ICH-specific sex-stratified data are scarce, national cardiovascular surveys have documented a high and, in some reports, rising prevalence of obesity, diabetes and metabolic syndrome among Emirati women, often exceeding that of men (27, 28). Cultural factors, including lower levels of leisure-time physical activity, barriers to participation in outdoor exercise, and delayed health-care seeking, may further amplify vascular risk in older women (27, 29, 30). These background data lend plausibility to our hypothesis that gendered social and behavioral patterns contribute to the unfavorable female trends. Targeted interventions could include women-centered hypertension and diabetes screening in primary care, culturally adapted programs to promote physical activity and healthy diet, and life-course approaches that integrate cardiovascular risk assessment into reproductive, menopausal and geriatric services. Strengthening sex-disaggregated stroke surveillance would also help to refine and monitor the impact of such strategies. Furthermore, our analysis of age-related trends in ICH DALYs reveals a concentration within the middle and older age groups (40 years and over), aligning with existing knowledge since middle age is commonly associated with an elevated risk for hypertension, diabetes, and lifestyle-related risk factors, pivotal contributors to ICH (31). In contrast, the striking shift in the ICH burden from older to younger and middle-aged populations in Saudi Arabia highlights varying points of concern. This epidemiological transition might reflect a rapid convergence of lifestyle risk factors traditionally seen in older populations, now increasingly affecting younger demographics due to urbanization, dietary transitions, and a sedentary lifestyle (32). Aggressive national policies addressing these modifiable risk factors could help curb this emerging trend and mitigate future disease burden.

Considerable disparities in the DALYs rate of ICH are evident among countries. Policymakers are encouraged to evaluate their nation’s specific attributes in this context, taking into account their standing in comparison to others for better-informed decision-making. The triggers for ICH often prompt inquiries, necessitating further exploration of age, period, and cohort effects. In response to these considerations, this study has centered on examining the ICH burden patterns globally and within BRICS-plus countries through the APC framework. Across all BRICS-plus countries, the longitudinal age curves were relatively flat through early adulthood and began to rise from approximately 40–44 years, with a much steeper gradient after 60–64 years. In most settings, the fitted DALY rates among those aged 70–74 years were several-fold higher than among individuals aged 40–44 years, and the curves either plateaued or increased more gradually beyond age 80. These patterns indicate that the acceleration of ICH burden is most pronounced in the young-old and middle-old age bands, rather than only in the very oldest groups. Biologically, aging predisposes individuals to ICH through various mechanisms. Arterial susceptibility to weakening due to cumulative exposure to hypertension, a prevalent risk factor that increases with age, could lead to spontaneous rupture of intracerebral vessels (33, 34). Additionally, age-associated loss of vascular integrity, manifested through amyloid angiopathy, and significant changes of immune system, are believed to exacerbate the functional impact of ICH (33, 35). From a public-health perspective, this supports prioritizing intensive blood-pressure control, diabetes and lipid management, smoking cessation, and careful use of antithrombotic therapies beginning in mid-life, together with age-friendly stroke education, fall-prevention strategies and rapid-response systems tailored to older adults. Another critical perspective is the global increase in life expectancy, resulting in a larger older population (12). This demographic shift underscores a transition in the predominant health issues from infectious to chronic non-communicable diseases, including cerebrovascular disorders.

In Brazil, the steepest decline in age-standardized DALY rates and the most pronounced negative period effects are likely multifactorial. Over the past three decades, the expansion of the Unified Health System and the Family Health Strategy has markedly improved access to primary care, antihypertensive treatment and secondary prevention for high-risk individuals (36, 37). National tobacco-control legislation, gradual reductions in salt intake, and large-scale public awareness campaigns around cardiovascular risk have contributed to declining population blood-pressure levels and improved hypertension control among older adults (36, 38). In parallel, the creation of specialized stroke units and the implementation of national stroke care guidelines have enhanced acute management and rehabilitation. Together, these structural reforms and risk-factor control policies provide a plausible explanation for Brazil’s 64.53% reduction in age-standardized ICH DALY rates, which exceeds the declines observed in other BRICS-plus countries with slower progress in universal health coverage or risk-factor management. China illustrates how rapid socio-economic development does not automatically translate into a lower ICH burden. Despite major gains in life expectancy, the country still has the highest age-standardized ICH DALY rate among BRICS-plus in 2021. Several factors likely underlie this pattern. First, the prevalence of hypertension remains high, especially in rural and older populations, and control rates have historically been low compared with many upper-middle-income countries (5, 31). Second, traditional dietary habits characterized by very high salt intake, combined with rising obesity, diabetes and persistent tobacco use in men, continue to fuel hemorrhagic stroke risk (5, 39, 40). Third, marked urban–rural and regional disparities in access to high-quality stroke care persist, so that many patients still present late or are treated in facilities without dedicated stroke units. The relatively flat period risk observed until the early 2000s coincides with rapid industrialization and urbanization, whereas the decline after 2006 parallels a series of national initiatives, including the 2009 health-care reform, the establishment of the China National Stroke Prevention Project and stroke-center networks, and the strengthening of hypertension and salt-reduction campaigns (39, 40). Nonetheless, the continued high level of ICH DALYs indicates that these reforms have only partially mitigated the accumulated burden of vascular risk factors, highlighting the need for further expansion of primary prevention and equitable access to acute stroke care. Period and cohort effects reflect significant shifts in healthcare delivery and public health interventions targeting cerebrovascular diseases in India, particularly after the launch of the National Programme for Prevention and Control of Cancer, Diabetes, Cardiovascular Diseases and Stroke (NPCDCS) in 2010, which scaled up community-based screening and management of hypertension and diabetes (41, 42). Despite this, the high absolute burden remains a concern, indicating insurance-based inequalities in access to healthcare services and delays in implementing effective hypertension screening programs in rural areas (41, 43). Cultural barriers, including lack of awareness and reliance on traditional medicine, still impact timely medical intervention in stroke management in India (44).

Epidemiological trends in the Russian Federation show relatively consistent period risks followed by a decrease, reflecting post-Soviet Union political–economic transitions affecting healthcare infrastructure and accessibility (45). The subsequent decline in period risk parallels the implementation of the Priority National Project “Health” initiated in 2005 and later federal cardiovascular programs that expanded primary-care coverage, subsidized antihypertensive drugs and reorganized emergency stroke pathways (46). These targeted reforms likely contributed to improved hypertension control and acute stroke management, thereby reducing the ICH burden over time. The initially stable and subsequently falling cohort effect in South Africa pointed to broad public health and stroke prevention campaigns gaining momentum gradually (47). The decline is likely aided by improved local healthcare services as part of the National Health Insurance reforms and advancements in public health afforded by increased government spending (48). However, challenges persist due to disparities in healthcare access influenced by socioeconomic inequality in South Africa (47, 49). The declining risks in Saudi Arabia, largely attributable to major health initiatives such as the Saudi Stroke Society’s prevention and management protocols, have led to improved early diagnosis and treatment efficacy for ICH cases (50). National health programs emphasizing lifestyle modifications and the reduction of hypertension and diabetes incidence continue to significantly impact ICH burden reduction (51). Similarly, significant reductions in period and cohort risks highlight enhanced health policies addressing cardio-cerebrovascular conditions in Egypt and the United Arab Emirates (52, 53). Government-imposed frameworks focusing on the early management of hypertensive crises contribute to effective ICH control (54). Cultural shifts toward healthier lifestyles also support these healthcare improvements. Despite intrinsic healthcare risks stemming from previously inefficient systems, current strategies are delivering optimistic momentum in decline, supported by educational campaigns in urban and rural sectors (55). Iran also demonstrates a decreasing trajectory in period effects. Recent healthcare innovations under the Health Transformation Plan have significantly improved the accessibility and quality of healthcare services (56). The country’s focus on non-communicable disease management and community-based programs has proven effective in reducing major risk factors (57). In Ethiopia, recent improvements correlate with the strengthening of the national healthcare system and prioritization of non-communicable disease control. The establishment of regional medical centers and community-based health interventions to control hypertension are the cornerstones of these results (58).

Compared with previous GBD 2021 report (59), this study provides several additional contributions. First, it focuses specifically on ICH in BRICS-plus countries, a group of large emerging economies that share rapid socio-economic change but differ markedly in health-system structures and cardiovascular prevention capacity. Second, by applying an age–period–cohort framework to GBD 2021 estimates, we go beyond simple trends in age-standardized rates and provide a more granular description of how aging, calendar time, and birth cohort dynamics jointly shape the ICH DALY burden in each country. Third, we explicitly integrate country-specific contextual information on primary care expansion, hypertension control programs, and major policy reforms when interpreting period and cohort effects, thereby linking model-based temporal patterns to plausible drivers in each setting. Finally, we translate these findings into concrete, regionally tailored recommendations for ICH prevention and control, which may be directly informative for policy and practice in resource-constrained health systems. Nevertheless, several limitations should be noted. First, GBD 2021 aggregates data from heterogeneous sources such as vital registration systems, hospital records and surveys, with variable completeness and quality across countries. As a result, ICH DALY estimates for some BRICS-plus members, particularly those with sparse primary data, are more heavily model-based and carry wider uncertainty intervals. Second, DALYs do not distinguish between incident events and disability due to differing severity or access to care, which limits our ability to disentangle changes in ICH incidence from improvements in survival and rehabilitation. Third, while the APC model is powerful, its estimates can be complex to interpret and are subject to the inherent identifiability problem, despite our use of the IE method to mitigate this issue. In addition, as with all APC analyses derived from age-period grids, residual timing and cohort-size biases may lead to modest artificial fluctuations in cohort profiles. To address these issues, we used broad five-year age groups, six equally spaced periods, a Poisson log-linear IE model, and a sensitivity analysis based on conventional 5-year averaged DALYs data (Supplementary Figures S3–S6), and the consistent results strengthen confidence in the observed trends. Finally, our analyses were conducted at the national level and did not account for within-country inequalities or individual-level risk factors, so the findings should be interpreted as providing a macro-level picture of the ICH burden and its temporal dynamics. Future work would benefit from high-quality stroke registries, subnational APC analyses and linkage of GBD estimates with empirical clinical and administrative data.

## 5 Conclusion

This study provides a comprehensive and up-to-date overview of the ICH DALY burden in BRICS-plus countries over the past three decades, highlighting substantial but heterogeneous progress. The persistently high, and in some settings increasing, ICH burden among low- and middle-SDI members underscores the need to priorities population-wide detection and long-term control of hypertension, reduction of excess dietary salt intake, tobacco and alcohol control policies, and equitable access to acute stroke care. In high-burden countries such as China, the Russian Federation, India, South Africa and Ethiopia, scalable priorities include community-based blood pressure screening, simplified treatment algorithms with affordable fixed-dose antihypertensive combinations, task-shared management in primary care, and further strengthening of regional stroke networks and referral pathways. Countries that have achieved marked declines, such as Brazil and Saudi Arabia, should consolidate successful primary care and cardiovascular prevention programs and extend them to underserved groups to avoid widening internal inequalities. Tailoring these evidence-based strategies to the demographic profile, health-system capacity, and socio-cultural context of each BRICS-plus member will be essential for further reducing the burden of ICH and narrowing international disparities.

## Data Availability

Publicly available datasets were analyzed in this study. This data can be found at: the datasets generated during and/or analyzed during the current study are available in the GBD Data Tool repository (http://ghdx.healthdata.org/gbd-results-tool). This public link to the database of GBD study is open, and the use of data does not require additional consent from IHME.

## Footnotes

1
https://ghdx.healthdata.org/gbd-2021

2
https://analysistools.cancer.gov/apc/
