# Supplementary material for: Epidemiological trends and age-period-cohort effects on intracerebral hemorrhage burden across the BRICS-plus from 1992 to 2021
Source: Front Neurol. 2026 Jan 12;16:1575324. doi: 10.3389/fneur.2025.1575324 (PMC12833510; doi:10.3389/fneur.2025.1575324)
Supplement: Supplementary file 1 [file Data_Sheet_1.docx]

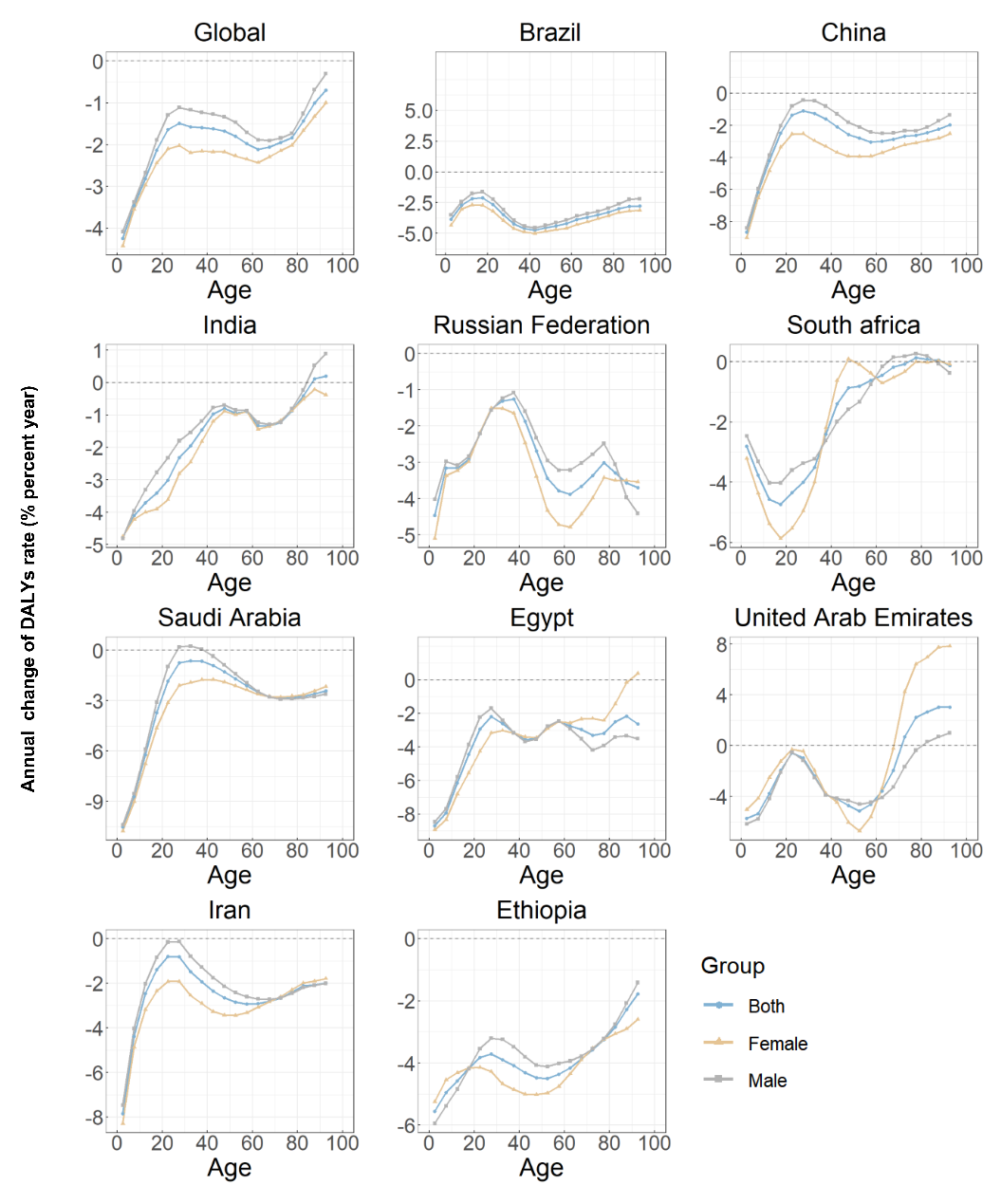


**Figure S1** Local drifts of intracerebral hemorrhage DALYs rate (estimates from age-period-cohort models) for 19 age groups (0-4 to 90-94 years) in global and BRICS plus, 1992-2021. The dots indicate the annual percentage change of DALYs rate (% per year). DALYs, Disability-adjusted life years.


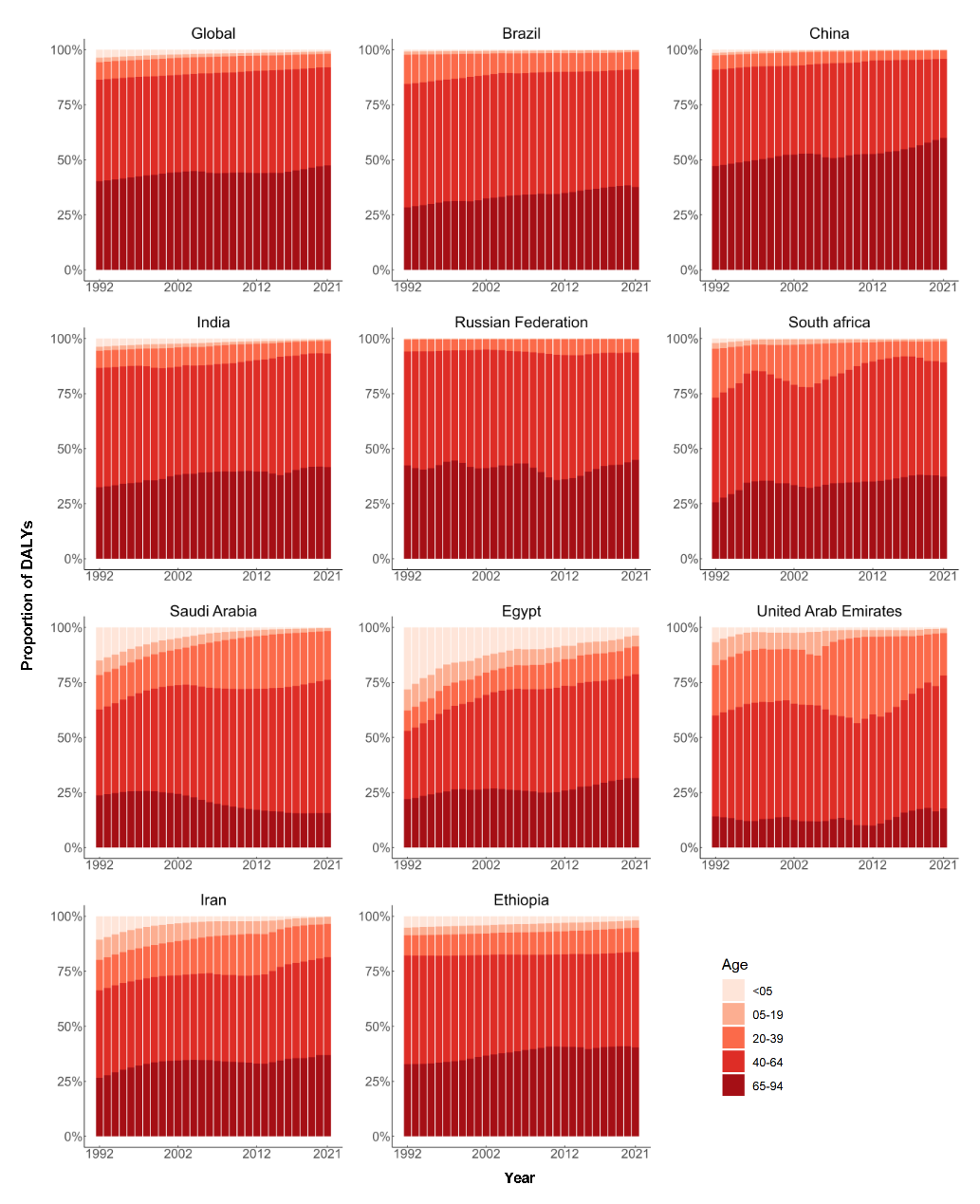


**Figure S2** Age distribution of disability-adjusted life years of intracerebral hemorrhage in global and BRICS plus, 1992-2021.


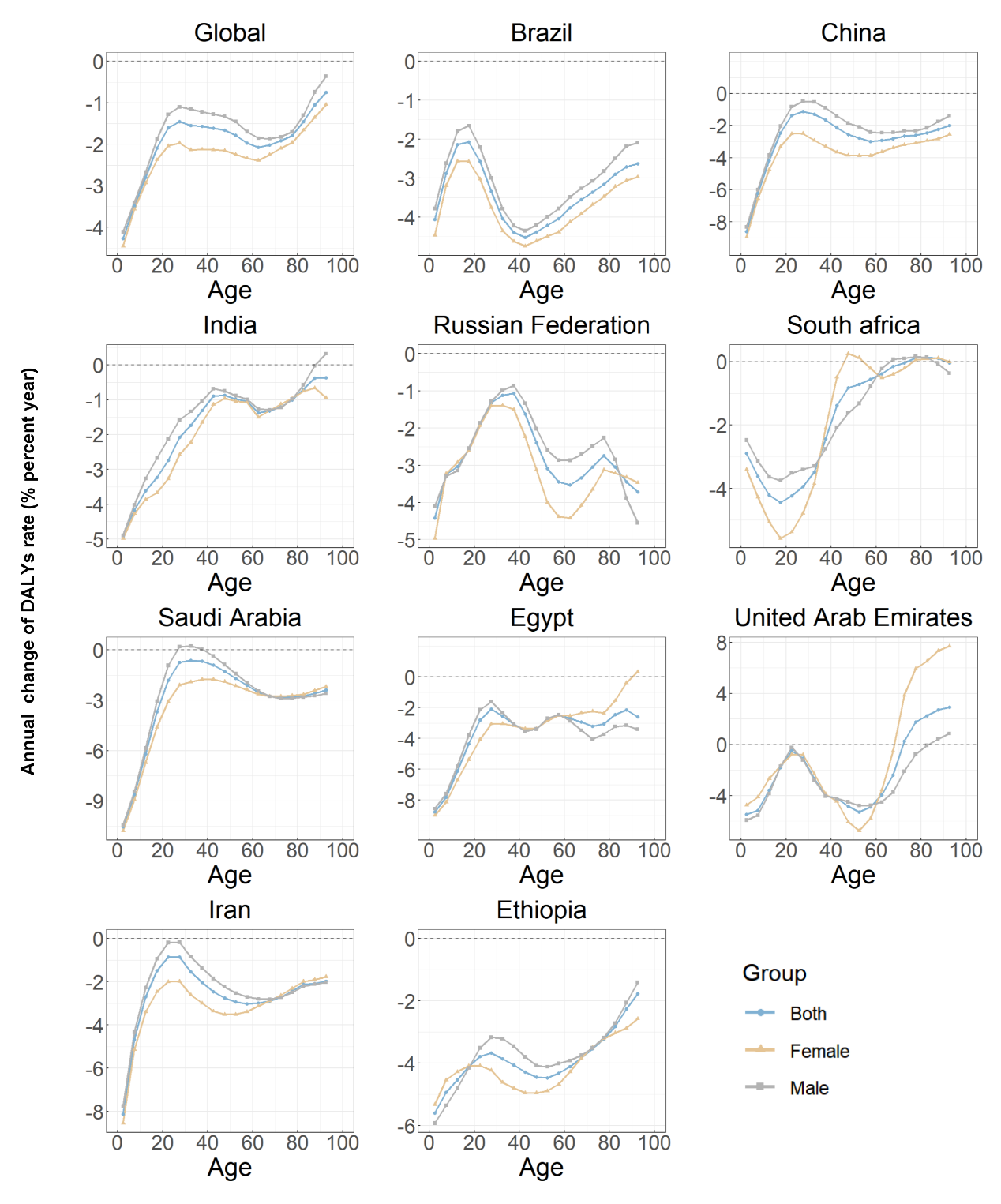


**Figure S3** Sensitivity analysis of local drifts of intracerebral hemorrhage disability-adjusted life years rate (estimates from age-period-cohort models) for 19 age groups (0-4 to 90-94 years) in global and BRICS plus, 1992–2021. The dots indicate the annual percentage change of disability-adjusted life years rate (% per year). The disability-adjusted life years of intracerebral hemorrhage and population data are centered on a 5-year average of six periods (1992-1996, 1997-2001, 2002-2006, 2007-2011, 2012-2016, and 2017-2021).


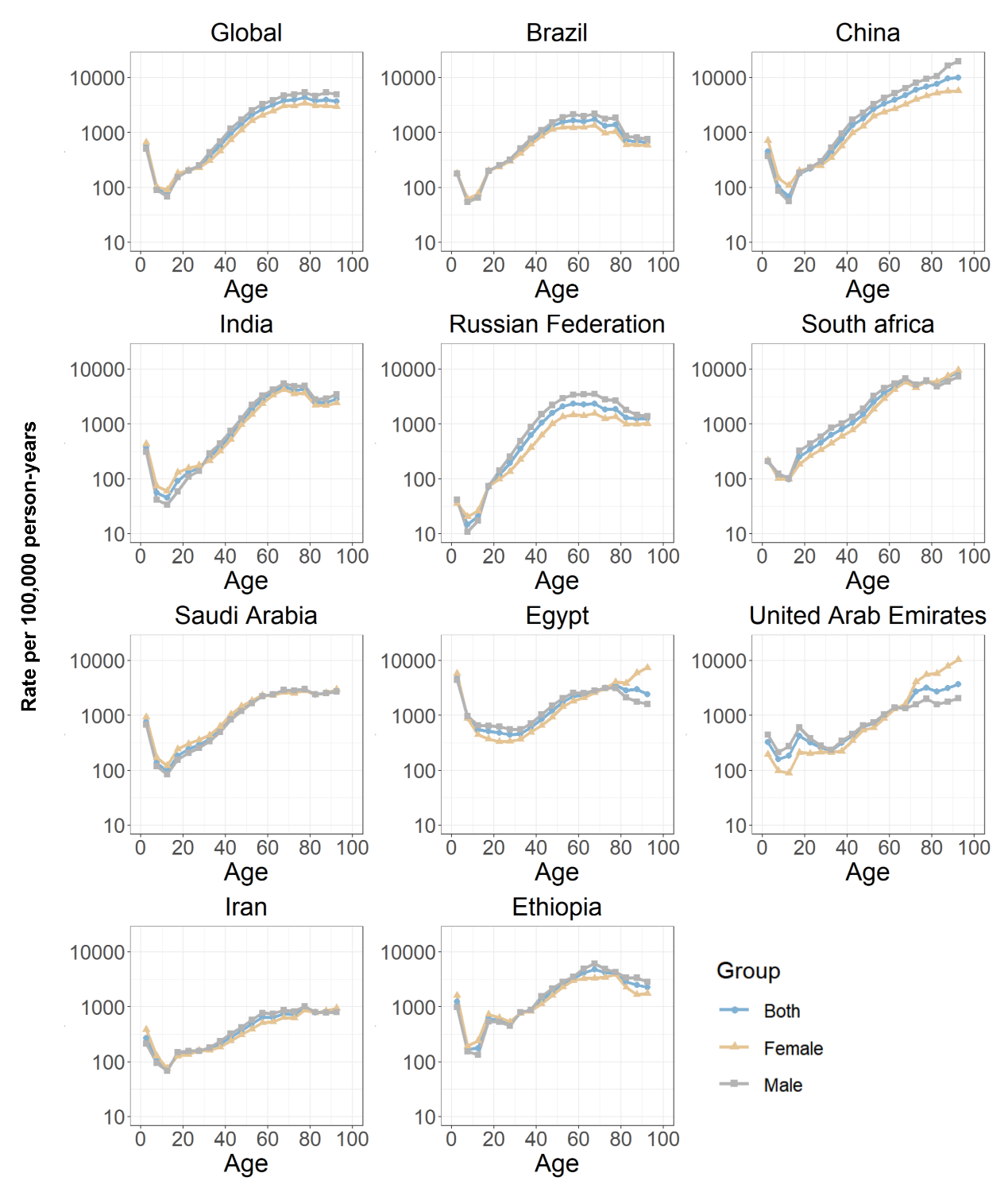


Figure S4 Sensitivity analysis of age effects on intracerebral hemorrhage disability-adjusted life years in global and BRICS plus. Age effects are shown by the fitted longitudinal age curves of disability-adjusted life years rate (per 100,000 person-years) adjusted for period deviations. The dots denote disability-adjusted life years rate. The disability-adjusted life years of intracerebral hemorrhage and population data are centered on a 5-year average of six periods (1992-1996, 1997-2001, 2002-2006, 2007-2011, 2012-2016, and 2017-2021).


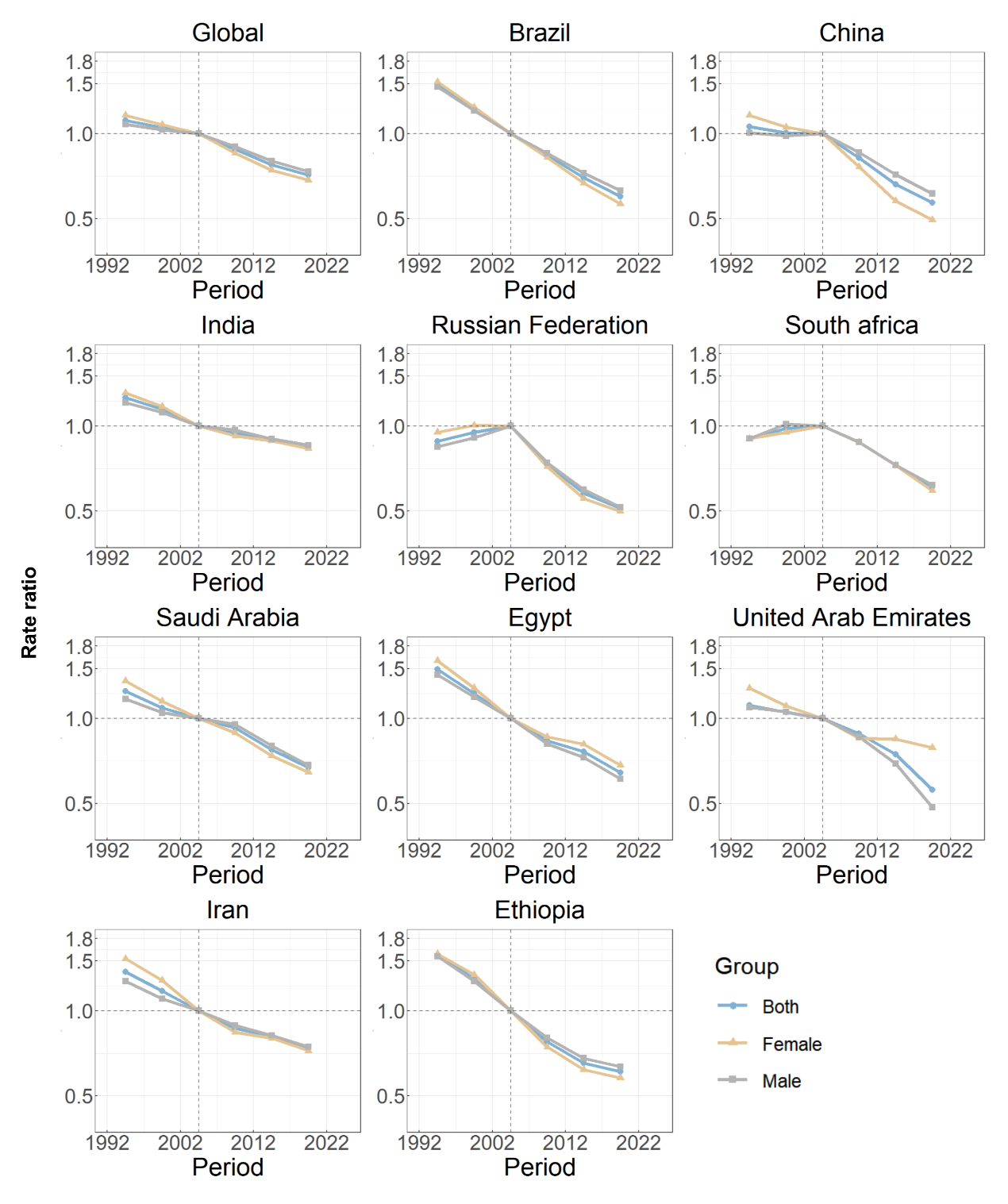


**Figure S5** Sensitivity analysis of period effects on intracerebral hemorrhage disability-adjusted life years in global and BRICS plus. Period effects are shown by the relative risk of disability-adjusted life years rate (disability-adjusted life years rate ratio) and computed as the ratio of age-specific rates from 1992-1996 to 2017-2021, with the referent cohort set at 2002-2006. The dots denote disability-adjusted life years rate ratios. The disability-adjusted life years of intracerebral hemorrhage and population data are centered on a 5-year average of six periods (1992-1996, 1997-2001, 2002-2006, 2007-2011, 2012-2016, and 2017-2021).


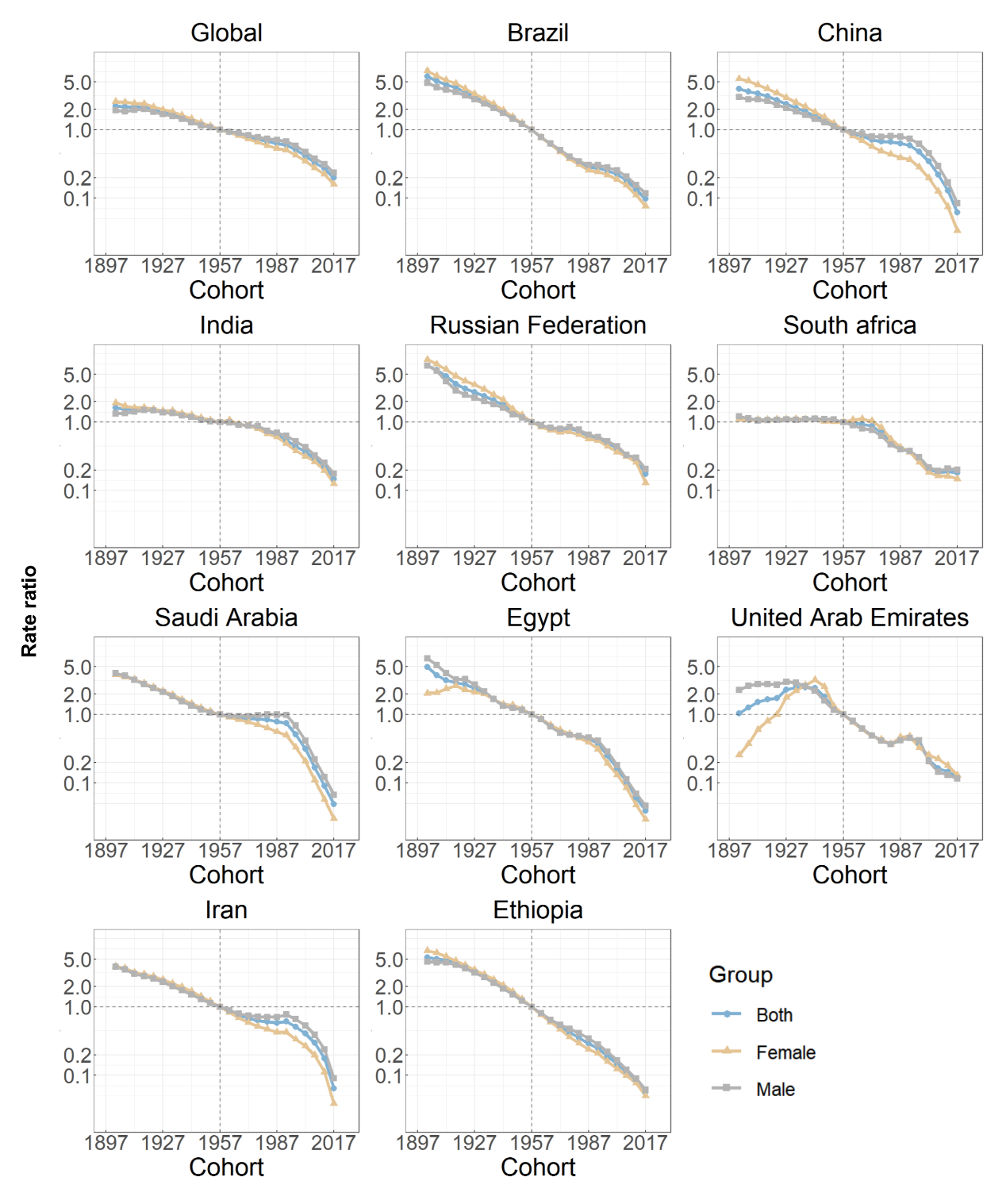


**Figure S6** Sensitivity analysis of cohort effects on intracerebral hemorrhage disability-adjusted life years in global and BRICS plus. Cohort effects are shown by the relative risk of disability-adjusted life years rate and computed as the ratio of age-specific rates from the 1902 cohort to the 2017 cohort, with the referent cohort set at 1957. The dots denote disability-adjusted life years rate ratios. The disability-adjusted life years of intracerebral hemorrhage and population data are centered on a 5-year average of six periods (1992-1996, 1997-2001, 2002-2006, 2007-2011, 2012-2016, and 2017-2021).
